# Characterization of the bacterial microbiome of *Rhipicephalus *(*Boophilus*) *microplus* collected from *Pecari tajacu* “Sajino” Madre de Dios, Peru

**DOI:** 10.1038/s41598-021-86177-3

**Published:** 2021-03-23

**Authors:** Jesús Rojas-Jaimes, David Lindo-Seminario, Germán Correa-Núñez, Benoit Diringer

**Affiliations:** 1 Facultad de Ciencias de la Salud, Universidad Privada del Norte, Av. El Sol 461, San Juan de Lurigancho, 15434 Lima, Peru; 2 DATAOMICS E.I.R.L., Lima, Peru; 3 Departamento Académico de Ciencias Básicas, Universidad Nacional Amazónica de Madre de Dios, Puerto Maldonado, Peru; 4 Incabiotec SAC, Tumbes, Peru

**Keywords:** Animal migration, Biodiversity, Ecological epidemiology, Ecosystem ecology, Microbial ecology, Molecular ecology

## Abstract

Ticks are arthropods that can host and transmit pathogens to wild animals, domestic animals, and even humans. The bacterial microbiome of adult (males and females) and nymph *Rhipicephalus microplus* ticks collected from a collared peccary, *Pecari tajacu*, captured in the rural area of Botijón Village in the Amazon region of Madre de Dios, Peru, was evaluated using metagenomics. The Chao1 and Shannon–Weaver analyses indicated greater bacterial richness and diversity in female ticks (GARH; 375–4.15) and nymph ticks (GARN; 332–4.75) compared to that in male ticks (GARM; 215–3.20). Taxonomic analyses identified 185 operational taxonomic units representing 147 bacterial genera. Of the 25 most prevalent genera, *Salmonella* (17.5%) and *Vibrio* (15.0%) showed the highest relative abundance followed by several other potentially pathogenic genera, such as *Paracoccus* (7.8%), *Staphylococcus* (6.8%), *Pseudomonas* (6.6%), *Corynebacterium* (5.0%), *Cloacibacterium* (3.6%), and *Acinetobacter* (2.5%). In total, 19.7% of the detected genera are shared by GARH, GARM, and GARN, and they can be considered as the core microbiome of *R. microplus*. To the best of our knowledge, this study is the first to characterize the microbiome of ticks collected from *P. tajacu* and to report the presence of *Salmonella* and *Vibrio* in *R. microplus*. The pathogenic potential and the role of these bacteria in the physiology of *R. microplus* should be further investigated due to the possible implications for public health and animal health in populations neighboring the habitat of *P. tajacu*.

## Introduction

Ticks are arthropods that can host a range of pathogens of other organisms and are one of the main vectors for vector-borne diseases^1^. *Babesia* sp. and *Rickettsia* sp. are pathogens frequently transmitted by ticks, whose detection and identification have been facilitated by molecular methods, particularly by the emergence of next-generation sequencing (NGS) techniques^2,3^. NGS techniques allow for (i) the precise characterization of the composition of complex microbiomes independent of the traditional culture techniques, (ii) the identification of pathogens, opportunists, probiotics, or commensals for the arthropod and/or host, and (iii) the calculation and comparison of the diversity and richness of microbiomes^4^. Although commensal and symbiotic bacteria have been identified by metagenomic studies in ticks^4,5^, these studies have focused on the microbiome with pathogenic potential from the veterinary and human perspective^6^. The microbiome biology in ticks still remains generally unexplored and neglected, and whether the microbiome has a neutral, harmful, or beneficial effect on the arthropod with regard to nutritional processes, adaptation, development, reproduction, or defense in adverse environments needs to be determined^6^. Furthermore, previous studies on *Ixodes pavlovskyi* have described *Rickettsia, Anaplasma, Ehrlichia,* and *Borrelia burgdorferi* as well as their impact on the vector and susceptible hosts^6,7^. Another study on *Dermacentor occidentalis* has identified an emerging pathogenic bacterium in humans called *Rickettsia philipii* as well as two new bunyaviruses^8^. The microbiome of *Rhipicephalus (Boophilus) microplus* has been characterized in cattle by pyrosequencing techniques^9^, while the pathogens *Anaplasma, Bartonella, Borrelia, Ehrlichia, Francisella*, and *Rickettsia* have been identified in ticks of the genera *Amblyomma* sp.*, Ixodes* sp.*,* and *Haemaphysalis* sp.^10^. Metagenomics has also been used to identify other infectious agents in *Rhipicephalus* sp., such as viruses, particularly nairoviruses that cause important diseases in humans^11^.

This study aims to analyze the bacterial microbiome in *R. microplus* collected from wild *Pecari tajacu* using metagenomics.

## Results

### Ticks collected from *P. tajacu*

**Figure 1:**
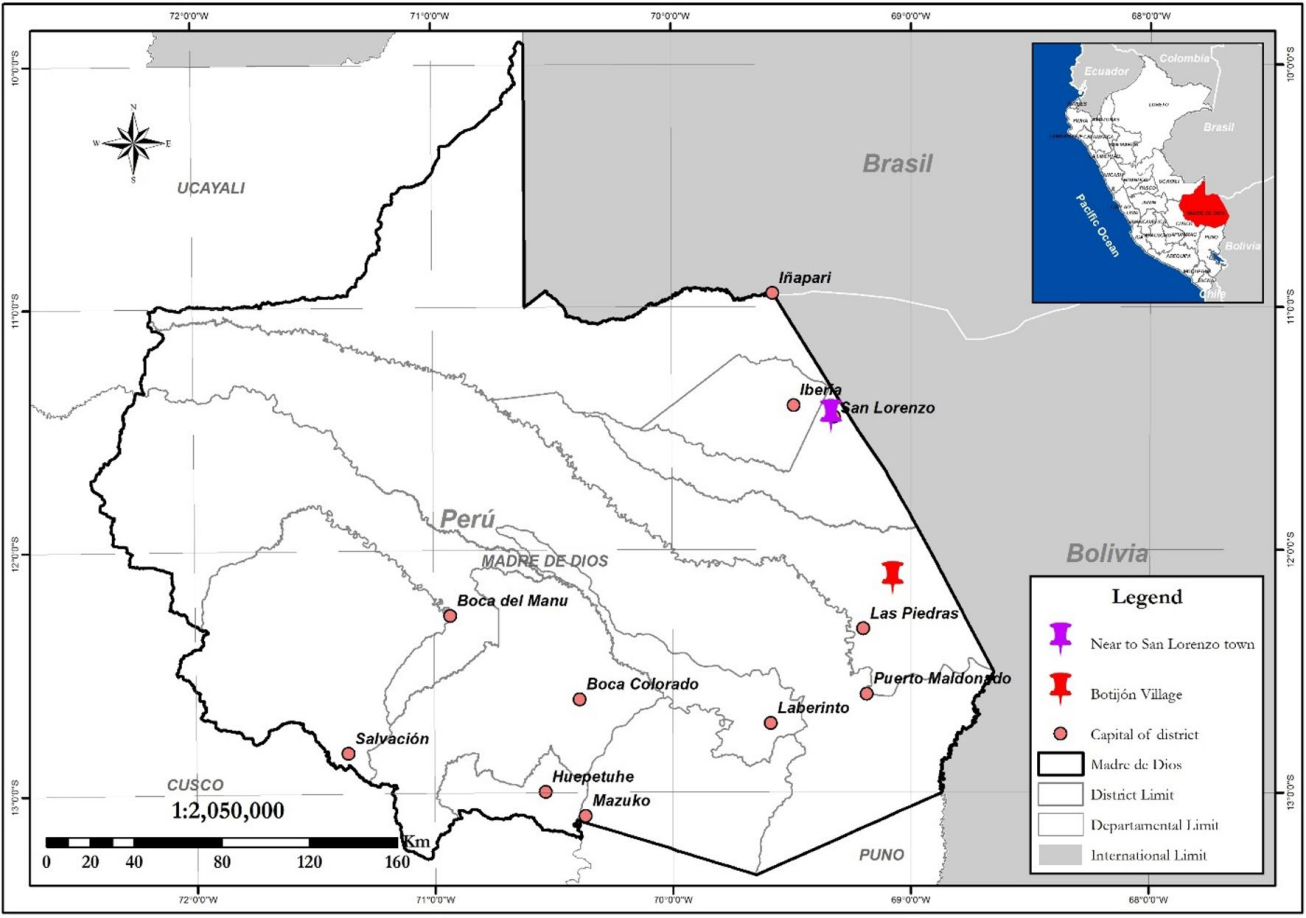
Botijón Village where samples were collected, and San Lorenzo town where cattle farming is practiced. This map was created with the Geoservidor https://geoservidor.minam.gob.pe/ edited with ArcGis 10.3.1 version 2015.

Taxonomic identification indicated that all the collected ticks in Madre de Dios (Fig. 1) belong to *R. microplus*^12^.

### Statistical values and diversity in the *R. microplus* microbiome

Microbiome analysis using the 16s-515F/16s-806R primers and amplicon sequencing on Ion Torrent PGM (Ion Personal Genome Machine System, THERMO FISHER SCIENTIFIC) generated a total of 117,192 raw reads (39,604 average) from the three analyzed samples^13–15^ (Table 1). After rigorous data curation, 55,805 high-quality sequences were retained with an average of 20,462 sequences per sample and an average length of 150 bp^16,17^. The maximum number of filtered sequences (26,549) was obtained from the female tick sample, which exceeded those found in male and nymph samples by 164.7% and 204.8%, respectively^18^. These sequences were assigned to 1075 total unique sequences corresponding to 185 abundant (< 0.005%) OTUs based on a > 97% identity cutoff for bacterial 16S rRNA genes^18^. At the individual sample level, the microbiome from nymphs surpassed that from females and males (221, 195, and 148 OTUs, respectively). Those OTUs were mainly identified as prokaryotes (99.89%) and to a lesser extent as unknown sequences (0.11%). At the taxonomic level, a total of 147 genera distributed in 99 families, 59 orders, 30 classes, and 12 phyla were detected.

**Table 1:** Statistical summary of the microbiota from *Rhipicephalus microplus*.

Sample size	GARH	GARM	GARN
Number of total sequences	56,059	32,524	28,609
Number of filtered sequences	29,084	18,103	14,200
Number of sequences assigned to OTUs	26,549	14,045	14,145
Number of OTUs	221	148	195
Phylum	10	6	9
Class	19	15	15
Order	43	29	34
Family	62	41	55
Genus	87	57	82
**Richness and diversity indexes**			
Chao1	375*	215*	332*
Shannon–Weaver	4.15*	3.20*	4.75*

*Significant differences (P < 0.01).

The SW index reflects the specific diversity of each sample, whose value increases as the number of different OTUs increases. In this study, the microbiome obtained from nymph tick samples showed a higher SW index than the female and male microbiomes. On the other hand, Chao1, the index that evaluates specific richness, showed that the number of expected OTUs decreased from 375 in GARH to 332 in GARN and 215 in GARM after the standardization of the sample size to 14,000 sequences. Statistical analyses of variance of the SW and Chao1 indexes in the GARH, GARM, and GARN samples showed significant differences (P < 0.01)^19–21^.

### Composition of the core and shared and individual microbiome from *R. microplus*

The comparative analysis of the composition of the microbiota from GARH, GARM, and GARN revealed that 19.7% out of the 147 genera found in *R. microplus* were common to the three groups. This shared community is considered as the core microbiota (Table 2). The percentages showed a decreasing proportionality in GARH, GARN, and GARM in relation to the non-shared bacterial genera. A higher percentage of shared microbiota was observed between GARH and GARN (9.5%) compared to that between GARM and GARH (3.4%) and between GARM and GARN (1.4%).

**Table 2:** Composition of the core microbiome according to *R. microplus* sex and stage*.*

Codes	Total	Genera
GARH GARM GARN	29 (19.7%)	*Streptococcus Brevundimonas Micrococcus Pseudomonas Corynebacterium Acinetobacter Staphylococcus Brachybacterium Ornithinimicrobium Lysobacter Brevibacillus Stenotrophomonas Other Bacillus Citrobacter Pluralibacter Janibacter Sphingomonas Salmonella Rothia Tepidimonas uncultured bacterium Nocardioides Paracoccus Aeromonas Vibrio Enterobacter Cloacibacterium Anoxybacillus*
GARH GARM	5 (3.4%)	*Trabulsiella Schlegelella Anaerococcus Actinomyces Neisseria*
GARH GARN	14 (9.5%)	*Blastococcus Sphingobacterium uncultured Comamonas Halomonas Dietzia Cupriavidus Tetrasphaera Granulicatella Thermus Deinococcus Kocuria Unassigned Rheinheimera*
GARM GARN	2 (1.4%)	*Brachymonas Lactococcus*
GARH	39 (26.5%)	*Phreatobacter Lawsonella Ruegeria Alteromonas Saccharopolyspora Bergeyella Enhydrobacter Prevotella Pseudorhodoferax Solobacterium Hydrogenobacter Morganella Gibbsiella Salinimicrobium Haematobacter Chryseobacterium uncultured Gemmatimonadetes bacterium Paenibacillus Gemella Cellvibrio Aquabacterium Abiotrophia Thiopseudomonas Craurococcus Peptoniphilus Myroides Bacteroides Shewanella Chroococcidiopsis Macrococcus Ensifer Gordonia Plesiomonas Salinicoccus Alishewanella Porphyromonas Fructobacillus Pantoea Lactobacillus*
GARM	21 (14.3%)	*Vulcaniibacterium Roseomonas Eikenella Enteractinococcus Mycobacterium Enterococcus Ilumatobacter Propioniciclava Hydrogenophilus Rubrobacter Peredibacter Georgenia Veillonella Thauera Chryseomicrobium Gemmatimonas Propionibacterium Diaphorobacter Cnuella Asticcacaulis Domibacillus*
GARN	37 (25.2%)	*Ochrobactrum Atopostipes Marinilactibacillus Alkalibacterium Paramesorhizobium Psychrobacter Ralstonia Sphingorhabdus Quadrisphaera Sphingobium Ottowia Exiguobacterium Brevibacterium Haemophilus Aerococcus Bradyrhizobium Luteimonas Flavobacterium Nannocystis Ruminococcus Novosphingobium Aeromicrobium Rubellimicrobium Acidovorax Pleomorphomonas Erythrobacter Sandaracinus Bordetella Serinicoccus Weissella Gardnerella Candidatus Alysiosphaera Klebsiella Leptotrichia Actinobacillus Isoptericola Atopococcus*

### Microbiota according to *R. microplus* sex and stage

**Figure 2:**
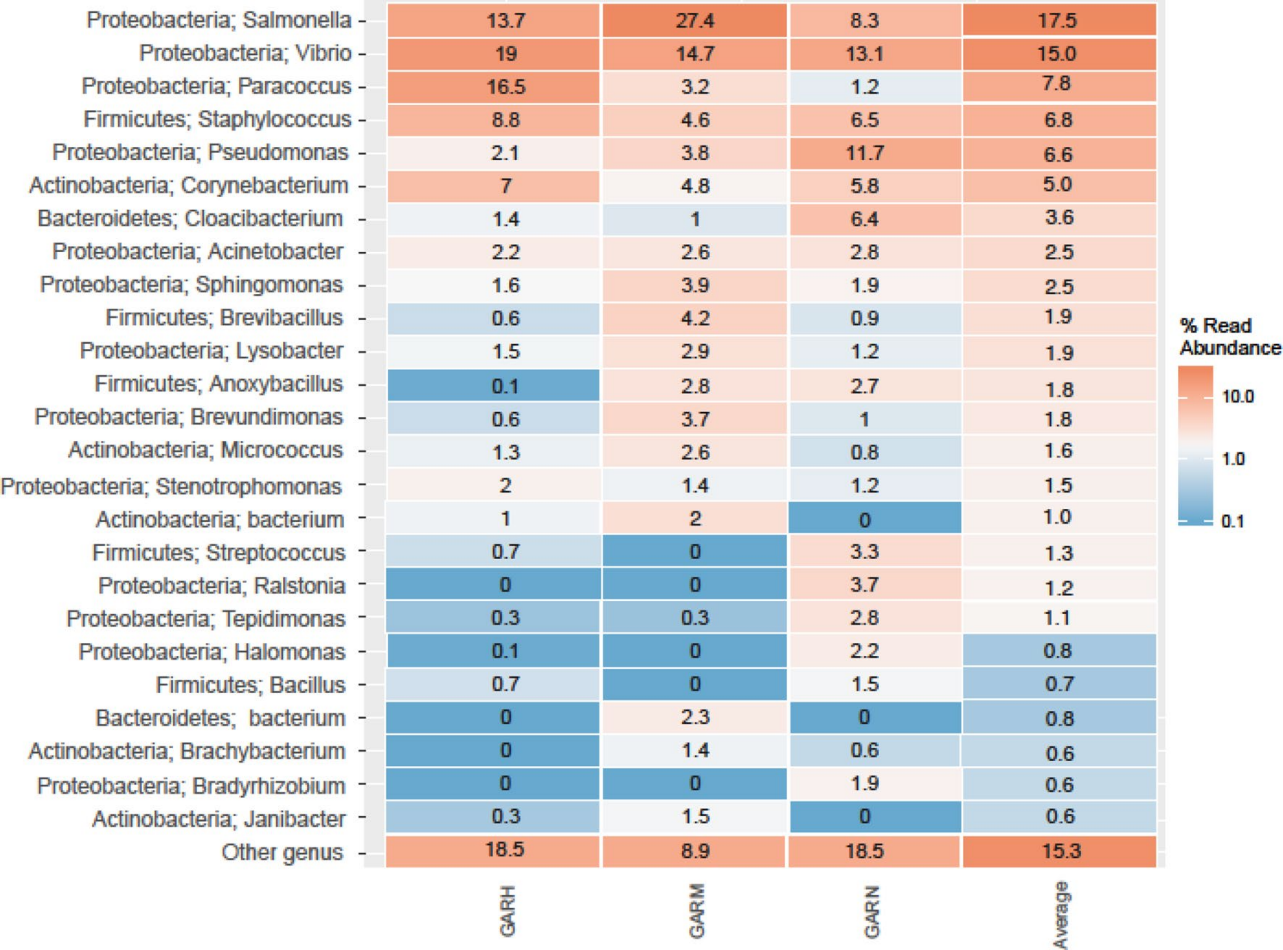
Microbiome abundance according to *R. microplus* stage and sex*.* (Rstudio version 3.2.3. https://cran.rstudio.com/bin/windows/base/old/3.2.3/).

Regarding the abundance of bacterial genera in *R. microplus*, *Salmonella* was the most abundant genus in GARM, while *Vibrio* was the most abundant genus in GARH and GARN, and *Paracoccus* was the second most abundant genus in GARH. On average, these were the most abundant genera in *R. microplus*, 17.5%, 15%, and 7.8%, respectively (Fig. 2).

## Discussion

The richness and diversity indexes revealed that the microbiota present in GARH and GARN exhibit greater bacterial genera diversity and richness than the microbiota in GARM. This is in agreement with previous studies on *R. microplus* that were collected from cattle^9^. Previous studies in male and female ticks of *Ixodes ovatus, I. persulcatus,* and *Amblyomma variegatum* have shown differentiated microbiome profiles both at the taxonomic and functional levels between sexes of the same tick species^22^.

A metagenomic study showed that the microbiome profile in ticks is related to metabolic processes and that their resilience and adaptability to the environment is related to their sex^22^. In addition, geographical location, temperature, humidity, species, sex, anatomical location, and type of diet have been shown to affect the microbiome of ticks^23–28^. In our study, although ticks were of the same species and were collected from the same host, significant differences were found in bacterial diversity and richness related to the sex and developmental stage of ticks.

Among the 147 different genera identified, the core microbiome that included the majority of the most prevalent genera stood out. Several of the identified genera within the core microbiome are known to be human pathogens (i.e., *Salmonella, Vibrio, Paracoccus, Staphylococcus, Pseudomonas, Corynebacterium, Cloacibacterium*, and *Acinetobacter*). In addition, a greater bacterial microbiome was shared between nymph and female ticks [14 (9.5%)] compared to that shared between male and female ticks [5 (3.4%)]. We suggest that these differences have a behavioral origin. Thus, female and nymph ticks are more prone to remain on the same host, whose microbiota impact on the tick gut microbiome, while male ticks frequently change hosts^22^. This hypothesis is supported by studies on other genera that reported higher relative abundance and alpha diversity in female ticks than in male ticks^22^. Additionally, it is necessary to consider that the role of nuclei bacterial genera and the species included in these may present different roles as pathogens or symbionts depending on whether they are found in the arthropod or in the vertebrate that hosts the arthropod.

The most prevalent genus among the three groups of ticks was identified as *Salmonella*, whose members cause gastrointestinal tract infection and dysentery and can lead to serious clinical conditions, especially in children^29^. The genus *Vibrio*, the second in abundance (15.6%), represents a finding of great interest as, to the best of our knowledge, this is the first study showing its presence in *R. microplus*. The genus *Vibrio* is a common commensal of aquatic arthropods and has a remarkable capacity for adaptation to the environment^30,31^. Its presence evinces the adaptation of this genus to the gastrointestinal system of *R. microplus*, which inhabits a jungle ecosystem. Many *Vibrio* are opportunistic pathogens of both arthropods and humans. Therefore, studying the virulence of the identified species is essential^30–32^. *Paracoccus*, the third most abundant genus (6.97%), is a coccobacillary bacterium that is typically present in a wide range of ecosystems^33^. *Staphylococcus,* with a prevalence of 6.63%, is mainly related to infections in soft tissues and has been previously reported in the gut of *R. microplus* and with a high prevalence in female *Amblyomma variegatum*^9,22^. *Pseudomonas* showed an abundance of 5.87% in *R. microplus*. In previous studies, the presence of this bacterial genus in *R. microplus* and in male *Amblyomma variegatum* with a high prevalence has been reported^9,22^. *Pseudomonas* has been suggested to be involved in the infection of soft tissues, including the tissues of the respiratory system^34,35^. The presence of *Corynebacterium,* with an abundance of 5.87%, is important because some *Corynebacterium* species produce the diphtheria toxin or can cause osteomyelitis^36^. In addition, this genus has been previously identified in eggs and male adults of *R. microplus*^9^. *Cloacibacterium*, with a prevalence of 2.93% in *R. microplus*, are gram-negative bacteria that proliferate in aqueous environments with high content of organic matter^37^. *Acinetobacter*, with an abundance of 2.53%, has been reported in a metagenomic study in *I. persulcatus, I. pavlovskyi,* and *Dermacentor reticulatus*^38^. *Sphingomonas*, the ninth most abundant genus (2.47%), includes non-fermenting and strictly aerobic gram-negative bacteria. Some species, such as *S. paucimobilis* and *S. wittichii*, can cause infections in immunocompromised patients^39,40^.

In contrast to the bacterial microbiome relevant to human health identified in our study, a previous study on bacterial diversity in *R. microplus* collected from cattle identified *Ehrlichia* sp.*, Coxiella* sp., and *Bartonella* sp.^41^. This indicates that the bacterial microbiome would also depend on the host parasitized by the ticks. Some bacteria, such as *Leptospira interrogans, Mycobacterium, Salmonella, Clostridium*, and *Pasteurella*, and tick genera, such as *Haemaphysalis, Dermacentor,* and *Amblyoma*, have been identified in the genus *Pecari*^42–44^. In our case, *R. microplus*, a tick that mainly parasitizes cattle^45^, was found in *P. tajacu* (*sajino*). *P. tajacu* was possibly tick infected due to the proximity of Botijón Village, where livestock farming is practiced. This highlights that ticks can infect cattle, *P. tajacu*, and humans, with the potential risks of pathogen transmission that this implies.

Regarding the role of bacteria in ticks, note that nonpathogenic microorganisms present in ticks could cause infections in humans and other animals. For example, ecological studies have shown that *Rickettsia, Francisella,* and *Coxiella*, which are considered vertebrate pathogens, can change their pathogenic role and have a mutualistic and symbiotic relationship with ticks^1^. Therefore, studying the interaction between the bacterial microbiota and ticks is of utmost importance for the control of pathogens and the development of the arthropod^1^. *Coxiella* sp. infects at least two-third of the ticks and is important for the survival of *Amblyomma americanum* and *Rhipicephalus* sp.^46,47^. Nonetheless, it has not been found in our study. *Coxiella* sp. and *Francisella* sp. are linked to the synthesis of vitamins necessary for the survival of ticks^48–50^. Likewise, other symbiotic bacteria, such as *Francisella, Rickettsia*, and *Rickettsiella*, have been reported^46^, with *Rickettsia* sp. and *Coxiella* sp. having become strict endosymbionts^1^. According to previous studies, the endosymbiont bacteria of a species of tick vary depending on the ecology and the number of ticks studied, for example, although in the case of *Coxiella* it was previously described *R. microplus* collected from cattle, previous studies that have demonstrated that the infection rates by *Coxiella* in *R. microplus* ticks are highly variable. A 2016 study evaluated *R. microplus* from Brazil, and found that only approximately 37% of the samples contained *Coxiella *^51^. In 2015 a study evaluated *Coxiella* in many species of ticks without finding the bacteria, one of the species of ticks evaluated was 3 *R. microplus* samples from Benin (west Africa) and did not find any *Coxiella *^52^. Therefore, the importance of our study is the finding of the new microbiome of *R. microplus* collected from *Pecari tajacu.*

The small number of ticks was justified by the fact that *R. microplus* ticks are not very common on the wild host *Pecari tajacu*; therefore, we could not collect a larger sample of ticks. On the other hand, we found interesting to test these ticks because We wanted to search for the microbiota of an exotic tick (*R. microplus*) infesting a mammal species native to the Amazonia (*Pecari tajacu*). Again, even though our sample was small, we have to highlight the interesting results We have found from these ticks.

Among the limitations of our study is the bacterial microbiome found in 5 females, 5 males and 2 nymphs of ticks collected from *P. tajacu*, which implies a bacterial microbiome representative of a specific circumstance and ecology. Therefore, studies with a greater number of samples could show a greater diversity of species and different percentages of bacterial abundance.

## Conclusion

In this study, we found a high bacterial diversity in female, male, and nymph *R. microplus* collected from *P. tajacu*. The greatest bacterial diversity and richness was found in females and nymph ticks compared to male ticks. The most frequent bacterial genera were *Salmonella, Vibrio*, and *Paracoccus*. This is the first bacterial metagenomic study performed in *R. microplus* collected from *P. tajacu* in the Peruvian jungle, and the presence of *Vibrio* is highlighted. This study lays the foundations for future studies on the importance of the role of the identified bacteria on arthropods and animal and human health.

## Material and methods

### Ethical aspects

This study was approved by the Oficina de Salud Pública y Medio Ambiente del Consejo Regional de Madre de Dios (Office of Public Health and Environment of the Regional Council Madre de Dios), Peru. Laboratory procedures for bacterial identification were conducted in accordance with the international guidelines for the use of animals in research and the standards of the Comité de Cuidado y Uso de Animales del Área de Investigación en Salud de la Junta del Consejo Regional de Madre de Dios (Animal Care and Use Committee of the Health Research Area of the Madre de Dios Regional Council Board). The study was carried out in compliance with the ARRIVE guidelines.

### Geographic location

The study was conducted in the outskirts of Botijón Village (12° 07ʹ 12.95ʺ S, 69° 04ʹ 31.47ʺ W; WGS 84, 267 m.a.s.l.), Tambopata province, Madre de Dios region, Peru (Fig. 1). The collection site corresponds to a forest area where hunting of wild animals is allowed. The average annual rainfall in the study area is 1600 mm^3^, and the average annual temperature is 25 °C. The area is in the tropical wet forest zone. During sample collection, the weather was hot and humid.

### Sample collection

A wild male of *P. tajacu* (*sajino*) was captured in Botijón Village in June 2012. The ticks from its abdominal region were collected 3 h after its sacrifice using forceps and were individually placed in 2 ml cryovials containing 96% ethyl alcohol. Cryovials were labeled with an identification code for the sampling site and the animal from which the sample was collected. Five male ticks, five female ticks, and two nymph ticks were identified. On sterile plates ticks were washed for 15 min in a solution 0.9% isotonic sterile sodium chloride saline followed by 15 min in a solution of 96% ethanol to remove surface contaminants. Excess solution was absorbed and ticks were air-dried prior to manipulation under sterile conditions. Each tick was individually cut in half lengthwise using sterile scalpels number 15.

### Taxonomic classification

Ticks were identified using taxonomic keys^12^ at the Laboratorio de Entomología del Instituto Nacional de Salud del Perú en Lima (Entomology Laboratory of the National Institute of Health of Peru in Lima).

### DNA extraction

Total DNA extraction from ticks was performed using Gentra Puregene Tissue kits (QIAGEN, Halden-Germany) according to the manufacturer’s instructions^13^ from pools for each tick sex and stage, i.e., GARH (females), GARM (males), and GARN (nymphs) pools.

### Metagenomics

To study the bacterial diversity and richness in the microbiota from *Rhipicephalus microplus*, the presence and quality of the extracted DNA was verified by PCR amplification of the 16S rRNA gene^14^ using the universal primers 27F (5′-AGAGTTTAGTCMTGGCTCAG-3′) and 1492R (5′-GGYTACCTTGTTACGACTT-3′) that generate a product of about 1500 base pairs (bp). All reactions were performed in 25 μl (total volume) mixtures containing 2.5 μl 10× buffer, 2.5 μl 25 mM MgCl_2_, 0.6 μl 10 mM dNTPs, and 2 U of Taq DNA polymerase (THERMO SCIENTIFIC). The PCR conditions were as follows: initial denaturation at 95 °C for 5 min followed by 35 cycles of denaturation at 95 °C for 30 s, hybridization at 55 °C for 45 s, elongation at 72 °C for 1 min, and a final elongation at 72 °C for 10 min. The PCR products were visualized by electrophoresis on a 1.5% agarose gel.

Total DNA extractions were analyzed by spectrophotometry (NANODROP EPPENDORF), and the samples with sufficient quality and quantity were shipped to MR DNA (Shallowater, TX, USA) and sequenced on the PGMplatform (Ion Personal Genome Machine System, THERMO FISHER SCIENTIFIC). Metagenomic analysis was performed on the PCR amplification products of the V4 hypervariable region of the 16S rRNA gene using the 515F/806R primers^15^.

### Analysis and processing of metagenomic data

The sequences generated by Ion Torrent were analyzed with QIIME v1.9.1^15^, where the initial sequences were processed based on filtering of barcodes ≤ 6 bp, Q25 quality scores, 150 bp sequence length, and chimera detection using usearch61^16,17^. High-quality sequences were assigned to operational taxonomic units (OTUs) with a 97% identity cutoff for bacteria. The final OTUs were classified taxonomically using the High Quality Ribosomal RNA Databases “SILVA” v132 database (https://www.arb-silva.de/). Likewise, unrepresentative OTUs ≤ 0.005% were filtered during analysis^18^.

Lastly, the final OTUs were processed to analyze the Shannon–Weaver (SW) alpha diversity index, Chao1 richness index, beta diversity (venn and heatmap), and taxonomic abundance (barplot) of the microbial communities using the phyloseq and ampvis packages with the statistical program RStudio version 3.2.3.^16,19,20^. Sequences shorter than 250 bp were removed. The obtained OTUs were then taxonomically classified using BLASTn and compared with a curated database derived from Greengenes, RPDII, and NCBI (www.ncbi.nlm.nih.gov^21^, http://rdp.cme.msu.edu^15^). The sequences were registered in Metagenomics Analysis Server “MG-RAST” ID: mgp95793; available at https://www.mg-rast.org/linkin.cgi?project=mgp95793.

## Abbreviations

GARH: Female tick
GARM: Male tick
GARN: Nymph tick
m.a.s.l.: Meters above sea level
PCR: Polymerase chain reaction
SW: Shannon–Weaver
OTU: Operational taxonomic units
PGM: Ion personal genome machine system
NGS: Next-generation sequencing
RNA: Ribosomal RNA
WGS: World geodetic system
SW: Shannon–Weaver
NCBI: National Center for Biotechnology Information
pb: Base pairs
